# Case of Placental Separation; Fatal Hemorrhage

**Published:** 1833-04

**Authors:** John Foote


### REPORTS
### FROM HOSPITALS AND MEDICAL INSTITUTIONS.

---

### *Case of Placental Separation; fatal Hemorrhage.* By John Foote, jun., Esq., L.A.S., A.M.B.S.

On Tuesday, the 26th of February, 1833, I was invited by Mr. Gerard Davis, a surgeon residing in Great Chapel street, Westminster, to accompany him to witness the autopsy of a woman, who had died undelivered. Messrs. Davis, D. O. Edwards, G. Pearse, and Gibbon, were present, as likewise the midwife who had been in attendance. The history of the case, as far as could be gleaned from the friends and the accoucheuse, was as follows:

Margaret Morris, ætat. thirty-five, a widow, but kept by an Irishman of the name of Welsh, residing 5, New Way, Westminster, obtained a card from a charity on which the addresses of two midwives were inscribed; she expected to be put to bed about the middle of March in the present year. She was the mother of six children, of which four are living; labours hitherto natural. She had a stand in the Broadway, where she was accustomed to sell fruit, supporting her basket by a band across the loins; owing to the vigilance of the new police force, she had latterly been obliged to keep moving about with her burden. Like the generality of those of her class, her habits were very intemperate.

After conception, she was admitted into the Westminster Hospital for abdominal dropsy, and, while there, she considered herself to have quickened.

On Saturday, the 23d instant, when about eight months and a half gone, she carried very heavy loads of coals and potatoes, and also drank a very large quantity of spirits. Within a few hours afterwards, she was taken ill, and lay down on the bed. On the Sunday afternoon, flooding came on to such an extent, that the blood soaked through the bedding, and deluged the floor. She also suffered from severe and unremitting pain in the left side of the abdomen, but unaccompanied by true labour-pains. The first midwife on the list was immediately sent for; but, ere she arrived, (four o'clock,) the hemorrhage had ceased, and the marks of it on the floor had been effaced. She was, however, informed of that circumstance. She made an examination, and found the os uteri undilated (as she said), so that she could not ascertain the nature of the presentation. She merely ordered her to take some hot tea, and hot brandy and water, and left her. The flooding occurred again very severely in about two hours, there being constant draining in the interim; the midwife was sent for a second time, but, when she came, the woman was insensible and evidently sinking. The man, Welsh, was immediately dispatched for the parish-doctor, but, as that gentleman was not in the way, he went for another surgeon, and when he got there, he found that the woman had been dead a quarter of an hour. She expired at a quarter past eight in the evening, all external hemorrhage having ceased by seven. During the whole time, there were not any labour-pains.

Autopsy. *External appearances.* Body generally pale; countenance much blanched; features calm; abdomen prominent, presenting an even sensation to the hand; a blood-stain on the left thigh.

*Internal.* An incision being made from the umbilicus to within three inches of the pubis, and then caused to diverge to either side, as far as the anterior superior spine of the ilium, some serous fluid was evacuated, and the uterus came fully into view. Its parietes were then cautiously incised, and the membranes, containing the liquor amnii in considerable quantity, exposed. These latter had not been ruptured, either by nature or by art; they were adherent throughout nearly their whole extent to the internal surface of the uterus, which was also considerably phlogosed. On the left side of the uterine cavity, a dark coagulum of blood, weighing between two and three pounds, was discovered, lying between the matrix and a thin flattened placenta, as large as an ordinary plate. The after-burden was nearly entirely separated from the uterus; the membranes being slit open, the os tincæ was ascertained to be dilated to the size of half a crown, and the head of the fœtus, a male, to have descended into the cavity of the pelvis, the vertex presenting, but not far enough to fill up the hollow of the sacrum. There was not any rupture either of the womb or umbilical chord; and the uterine veins, some of which were traced to a considerable extent, were healthy. The pelvic bones of the mother appeared to be well-formed, and sufficiently capacious to allow an infant of the ordinary size to pass. There was not any undue enlargement of any part of the fœtus, which might obstruct the natural termination of parturition. The liver presented the usual appearances of that of the drunkard. The other cavities were not examined.

There can hardly be a doubt in the minds of any one that this woman lost her life from gross mismanagement and ignorance on the part of the midwife; and neither should I hesitate in affirming, that, had a properly-educated practitioner been in attendance, she would not have perished. As she had previously borne six children, her labours hitherto natural, as there was not any deformity either of the mother or child, and the presentation natural, it may be reasonably surmised that, had she been properly treated, labour would have come on, the hemorrhage would have been arrested, and the mother saved, although the child, in all probability, would have been lost. An obstetric pupil of only a year's standing ought, and I doubt not would, have treated the case better: he would have dilated the os uteri, ruptured the membranes, and induced labour. The mere ruptures of the membranes might have sufficed to prevent the recurrence of hemorrhage, and the case might then have been left to the efforts of nature, or else labour expedited by the secale cornutum. If it be urged, that the symptoms present when the midwife came the first time did not warrant manual interference, it must be admitted that the woman ought not to have been left after the occurrence of so formidable an attack of flooding, especially as there was constant draining, inasmuch as it was very liable to recur, and cause syncope and death. But it must be apparent, that, in the case before us, it was the duty of the midwife to have ascertained the nature of the presentation by dilating the os uteri sufficiently, as it might arise from a placenta presentation, and then active measures are imperatively necessary. It does not appear, from what I have heard both from the midwife and other persons, that any thing was done for the woman's relief, except the exhibition of tea and brandy and water in the first instance, and sending for a medical man when she was in articulo mortis; so that the midwife's conduct was reprehensible throughout. Had a coroner's inquest been held, can a doubt be entertained as to the nature of the verdict?

Cases similar in the result are not of unfrequent occurrence in the hands of midwives, a race of beings frequently extremely ignorant, and only capable of managing a case successfully when nature does the business to their hands. Some, however, have sufficient common sense to induce them to send for other advice, and for an individual more competent to conduct the case, when it becomes too onerous for them, and they are anxious to get rid of the responsibility; but here, as in many others, the assistance of a surgeon was not required until the time had passed when he might have been of service. For these reasons, and others which might be adduced, the sooner the midwife is prevented practising, the better it will be for science and the public weal, notwithstanding the reveries of a worthy but eccentric knight, who, although a good surgeon, is most certainly practically unacquainted with obstetricy. To my knowledge, there are lecturers on midwifery who do not hesitate to teach women in private the art and science they profess, while they rave at and run down Sir A. Carlisle for his whim-whams in public: such conduct alone is sufficient to stamp the persons I allude to with disgrace, and they richly merit to be held out for public contempt; but it is an ungracious task to point out the errors of our brethren, and I decline it.

36, *Tavistock street, Covent Garden.*

